# Autochthonous liver cystic hydatid: Past or actual French shepherd's disease?

**DOI:** 10.1016/j.idcr.2020.e00843

**Published:** 2020-05-26

**Authors:** Florent Darriet, Nadim Cassir, David J. Birnbaum, Jérôme Soussan, Estelle Menu, Stéphane Ranque, Coralie L’Ollivier

**Affiliations:** a IHU-Méditerranée Infection, Marseille, France; b Aix-Marseille Université UM63, Institut de Recherche pour le Développement IRD 198, Assistance Publique – Hôpitaux de Marseille (AP-HM), Microbes, Evolution, Phylogeny and Infection (MEΦI), Marseille, France; c Department of Digestive Surgery, Hôpital Nord, Assistance Publique-Hôpitaux de Marseille, Aix-Marseille University, Marseille, France; d Department of Radiology, North University Hospital, Chemin des Bourrely, 13015, Marseille, France; e Aix Marseille Univ., IRD, AP-HM, SSA, VITROME, IHU-Méditerranée Infection, Marseille, France

**Keywords:** Hydatidosis, French, Indigenous, Shepherd

## Abstract

Autochthonous hydatidosis in France and western Europa are uncommon since the beginning of the 21st century. We report here an authentic indigenous cystic echinococcosis case in a French shepherd. The risk of remerging pathology should not be neglected and measures to interrupt parasite transmission are still relevant.

## Introduction

*Echinococcus granulosus* is a cestode of the Tæniidæ family which mainly infects domestic dogs as a permanent host. It is transmitted to a wide range of intermediate-host domestic ungulates (e.g., sheep, goats, cattle, camels, and cervids) which harbor the hydatid cysts [1]. Cystic echinococcosis (CE) is an important zoonotic parasitic infection that causes morbidity and mortality in humans. Humans become infected by ingesting eggs from contaminated food or water, or from direct contact by handling infected dogs or their egg-containing feces [2]. CE is usually asymptomatic: the development of the larvae occurred most commonly in the liver (50–70 % of cases) [3], and less commonly in the lungs (20–30 %), spleen, kidneys, brain and heart [4]. Nevertheless, the infection may become symptomatic if the cysts either break or exert a mass-effect.

Hydatidosis has a worldwide distribution from tropical to Artic areas [5]. It typically occurs in poor pastoral regions, in which sheep or other livestock are raised and in which dogs are kept, for herding or property guarding, in close proximity to households. In Europe, published data suggest that the prevalence is rather high in some regions in Italy, Spain and eastern Europa [6]. In France, the annual incidence of human CE cases of has been estimated at 260 hospitalized patients [7]. The southeast area, especially Provence-Alpes-Cote d’Azur and Corsica regions, is the historically CE endemic area known in France. Because no mandatory monitoring program of human CE infections exists in France, the relative proportion of imported or indigenous cases is impossible to asses. Indeed, this area is relatively close from 3 hyperendemic CE areas, namely Spain, Italy and Northern Africa. Apparently, French autochthonous cases among shepherds became clearly uncommon without report since the end of the 20th century, so that local CE seemed to have disappeared. Here, we report an indigenous CE case in a French shepherd.

## Case report

**Fig. 1:**
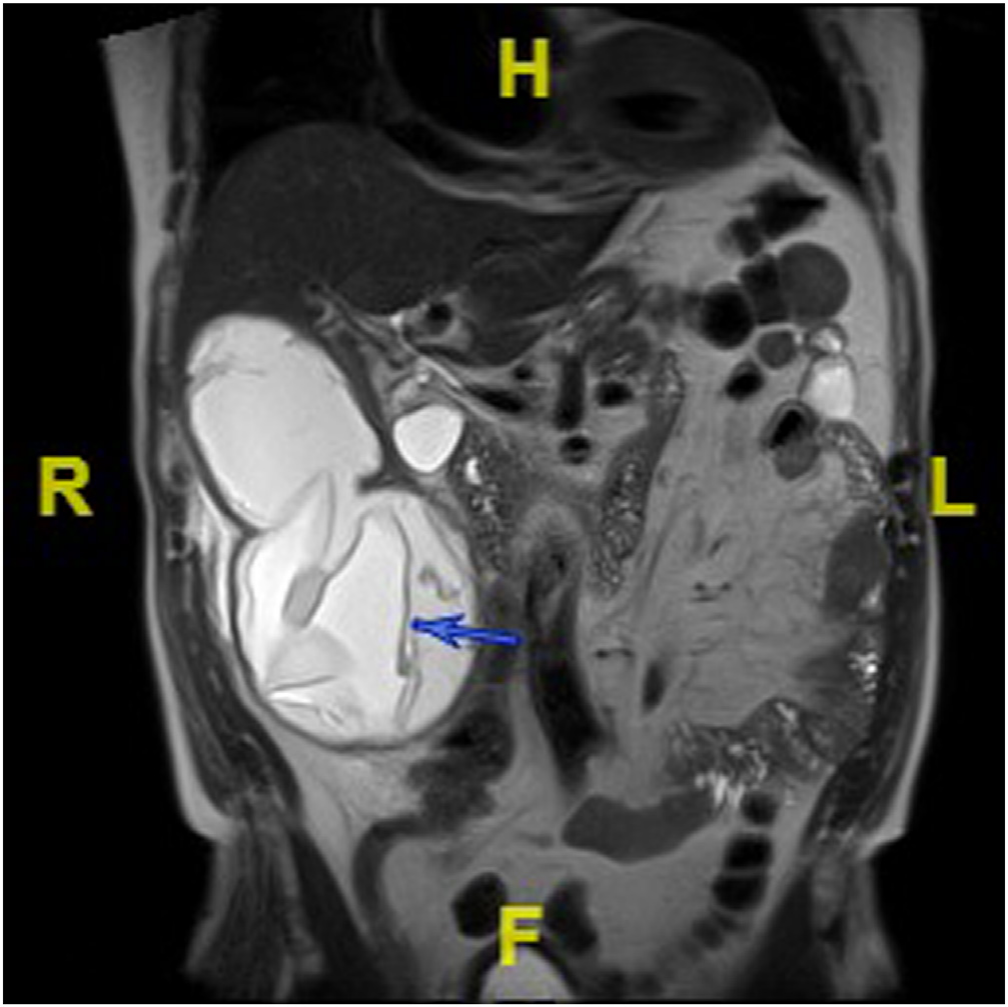
Magnetic resonance imaging. Coronal section in T2-weighted pulse sequence. Cystic bilobate lesion with exophytic part. WHO Classification: CE3a: Liquid cyst with detached membrane appearance.

In June 2017, a 78-year-old Frenchman, was hospitalized for episodic pain in the right hypochondrium for one month. His medical history included prostate local resection in 2013, inguinal hernia and daily both alcohol drinking and tobacco smoking. The patient was a traditional shepherd who never traveled outside the South-East of France. He had daily contact with sheep, sheepdogs, donkeys, horses and cats. Physical examination revealed a painful mass in the right hypochondrium. Computed tomography and magnetic resonance imaging scan showed three liver cysts, located in segments IV, V and VI (Fig. 1), that were classified following the WHO standardized US classifications in two CE5 cyst sized 5 cm and a bi-vesicular CE3b cyst sized 10 cm, respectively. No remarkable biological parameters were found, with the exception of a 78 mg/L C-reactive protein level (N < 5 mg/L). Hydatidosis serology was negative.

**Fig. 2:**
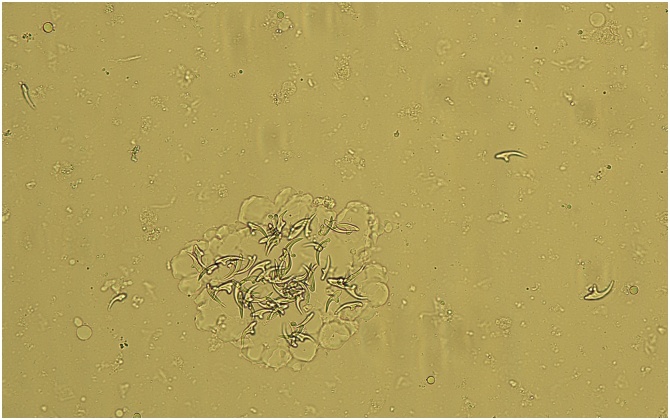
Puncture fluid with typical hooklets of *Echinococcus granulosus.*

Based on CT-scan images, a diagnosis of liver hydatid cyst was retained. The patient received albendazole (ABZ) 400 mg twice per day for six months. ABZ was stopped temporarily during a course of 2 months because of lymphopenia. The follow-up showed a suspected pre-rupture of the largest cyst: the serology switched to positive, thus precipitating the decision of surgical treatment by resection of the protruding dome in March 2018. The postoperative biological examination of the puncture fluid revealed scolex and typical hooklets (Fig. 2). Molecular test based on the cytochrome C oxidase subunit 1 nucleotide sequence has made it possible to identify *Echinococcus granulosus sensu stricto* genotype G1 (99 % on gene bank: HF947568.1). ABZ treatment was continued up to May 2018. At 2 months of follow-up, the patient had no pain and CT-scan images showed only the 4 cm calcified cyst in segment IV.

## Discussion

In western Europe, helminthiasis are usually diagnosed in migrants or travelers [8] as indigenous cases are scarce. Human CE cases in France are not an exception to the rule. Umhang et al. [9] found low prevalence of *E. granulosus* (4/100,000 sheep and 3/100,000 cattle) at slaughterhouses in southern France. Despite this fact, only one human case [10] has been reported from this major historical endemic area within over the past two decades. Also, human CE are usually imported cases; mainly from Spain, Italy and Northern Africa. This present report highlights the fact that autochthonous CE is still present, especially among shepherds who practice transhumance in the mountain pastures that is, moving livestock in a seasonal cycle typically to lowland in the winter and highlands in the summer. The carcasses of sheep that die on the way can be available for the sheep dogs.

The national survey of Umhang et al. [9] demonstrated that CE is endemic in livestock. Furthermore, rare human cases have been reported in France [10,11] in recent years. All these data converge towards the evidence of the parasite’s development at different stages of its life cycle in French rural area. Nevertheless, there is no large data available on CE in sheepdog. It is admitted that key to success of CE control is the removal of worm biomass in dog, by interrupting the parasite life cycle and reducing human exposure [12]. Praziquantel (5 mg/kg), the anthelminthic agent used to deworming dogs has neither a residual nor an ovicidal effect, and should thus be preferably administered each six weeks to prevent egg output. A health education program on hygiene, deworming and dogs contact targeting shepherds would also be of interest in implementing in CE endemic areas in France.

The genotypes G1, G2 and G3 of *E. granulosus* stricto senso (recovered from sheep) are endemic in south of France. In line with these data, the genotype G1 was involved in this patient’s cyst. The standardized classification of CE proposed by the WHO-Informal Working Group on Echinococcosis (WHO-IWGE) [13] defined six cyst stages assigned to three clinical groups (Table 1). This classification provides a rational basis for choosing an appropriate CE treatment scheme and follow-up, *i. e*. surgery, percutaneous treatment such as PAIR (puncture, aspirate, injection, re-aspirate), albendazole chemotherapy or ‘watch & wait’ (WW). Our patient presented with 3 cysts, two classified CE5 and one CE3b. Conventionally, WW is appropriate for uncomplicated inactive CE5 cyst [14], whereas management of transitional CE5 cyst is unclear. Rinaldi et al. showed that several months ABZ treatment induces CE3b cysts inactivation. In this present case, the patient was treated by ABZ during 6 months. However, during the follow-up, evidence of cyst’s pre-rupture, including the seroconversion of both ELISA and immune-blotting tests, posed a threat of secondary hydatidosis and anaphylactic shock triggered by the release of viable content of the CE3b cyst. It is important to note that this 10 cm long cyst was at risk of rupture [14] and that the recommendations are to promote surgical treatment and to avoid PAIR procedure [15]. Therefore, the patients treated by a long course of ABZ therapy should be regularly followed-up to assess both adverse events (*i.e*. alopecia, liver toxicity, and agranulocytosis) and cyst leakage.

**Table 1:** Simplified classification of liver hydatic cysts according to the WHO informal working group [13].

Stages of cysts	Clinical group	Imaging (ultrasound) features
CL	active	Unilocular cyst, anechoïc, no wall depicted
CE1		CL characteristics + “snow flake” signs
CE2		Multivesicular (daughter) cysts associated with “wheel-like” “rosette-like” or “honeycomblike” structures
CE3^a^	transitional	Detachment of laminated membrane + “water-lily sign”
CE4	inactive	Heterogenous hypoechoic cyst
CE5		CE4 characteristics + a thick calcified wall

a CE3 cysts have been further divided into CE3a (active or inactive) and CE3b (active).

This patient’s case, associated with the other reported indigenous cases in western Europe, highlight that CE risk persists in these rural areas. Therefore, some recommended measures to interrupt parasite transmission are still relevant, including: controlling livestock slaughtering, proper disposal of offal particularly viscera, regular deworming of dogs with praziquantel and vaccination of intermediate hosts [12]. Autochthonous or imported hydatidosis in France should not be neglected. Indeed, the management of patients with CE requires a skilled and expert medical staff.

## CRediT authorship contribution statement

**Florent Darriet:** Writing - original draft. **Nadim Cassir:** Resources, Visualization. **David J. Birnbaum:** Resources. **Jérôme Soussan:** Resources. **Estelle Menu:** Resources. **Stéphane Ranque:** Supervision. **Coralie L’Ollivier:** Writing - review & editing.

## Declaration of Competing Interest

All authors declare any conflicts of interest or sources of funding
